# Cough-Induced Bilateral Internal Carotid Artery Dissection Complicated With Large Vessel Occlusion and Malignant Middle Cerebral Artery Syndrome

**DOI:** 10.7759/cureus.70252

**Published:** 2024-09-26

**Authors:** Dodik Saggurthi, Eluzai Hakim

**Affiliations:** 1 Department of Stroke Medicine, University Hospitals Dorset NHS Foundation Trust, Bournemouth, GBR

**Keywords:** alteplase, bilateral internal carotid artery dissection, large vessel occlusion, malignant mca syndrome, mechanical thrombectomy (mt), rare cause of stroke, stroke thrombolysis, young adult ischemic stroke

## Abstract

Cervical artery dissection (carotid artery and vertebral artery) is one of the important causes of cerebrovascular accidents, particularly in younger patients without traditional vascular risk factors. Coughing caused by respiratory tract infections can, in rare cases, lead to a vessel wall tear, potentially resulting in embolism. We present a 55-year-old man who came to the hospital with transient episodes of left arm weakness, dysarthria, and facial numbness, which progressed to left hemiparesis in three hours. A non-enhanced brain CT scan and cerebral angiography showed thrombus in M1 segment of the right middle cerebral artery (MCA) with extensive occlusion of the right internal carotid artery (ICA) with marked dissection of the cervical portion of the left ICA. He was initially treated with intravenous thrombolysis with alteplase, according to the hospital's protocol. His symptoms improved temporarily but he later developed a thrombus in the same vessel complicated by malignant MCA. We discuss the identification of carotid artery dissection, escalation, and management of the patient.

## Introduction

Cervical artery dissections, which include the carotid and vertebral arteries, can occur at any age. They constitute 2% of all strokes and 25% of strokes in individuals under 50 years old [1]. Coughing due to respiratory tract infections can rarely cause internal carotid artery (ICA) dissection [2]. Additionally, a history of migraines, especially migraines with aura, is consistently associated with an increased risk of cervical artery dissection [3]. Here, we discuss a patient with a cough-induced extracranial ICA dissection complicated by a malignant middle cerebral artery (MCA).

## Case presentation

A 55-year-old male patient presented to the emergency department (ED) of a district general hospital exhibiting symptoms consistent with a stroke and potential eligibility for thrombolysis with alteplase. He experienced transient episodes of dense left arm weakness lasting about a minute, dysarthria lasting five to 10 minutes, and left cheek numbness, with all symptoms occurring within a span of three hours. The patient reported a severe coughing fit prior to the onset of these symptoms, which were preceded by a three-week history of chest infection and cough. He had a history of migraine and had quit smoking 12 years ago with no history of diabetes, hypertension, or ischemic heart disease. Physical examination revealed a blood pressure reading of 121/84 mmHg and a National Institutes of Health Stroke Scale (NIHSS) score of 1 for left upper limb drift on the first assessment. His neurological symptoms worsened in the next 10 minutes, with the NIHSS score rising to 8, accounting for left facial palsy, left arm weakness, limb ataxia, and dysarthria. Full blood count and renal and liver profiles were normal. The connective tissue disease screen, including immunoglobulins G, M, and A, anti-nuclear antibodies, anti-dsDNA antibodies, anti-Sm antibodies anti-Rib-P antibodies, anti-PCNA antibodies, anti-U1-snRNP antibodies, anti-Ro antibodies, anti-La antibodies, anti-Scl-70 antibodies, anti-centromere antibodies, anti-fibrillarin antibodies, anti-RNA polymerase III antibodies, anti-Jo-1 antibodies, anti-Mi-2 antibodies, anti-PM-Scl antibodies, and lupus anticoagulant, was negative. A fasting lipid profile showed slightly elevated cholesterol levels and normal glycosylated hemoglobin (HbA1c) levels.

**Figure 1:**
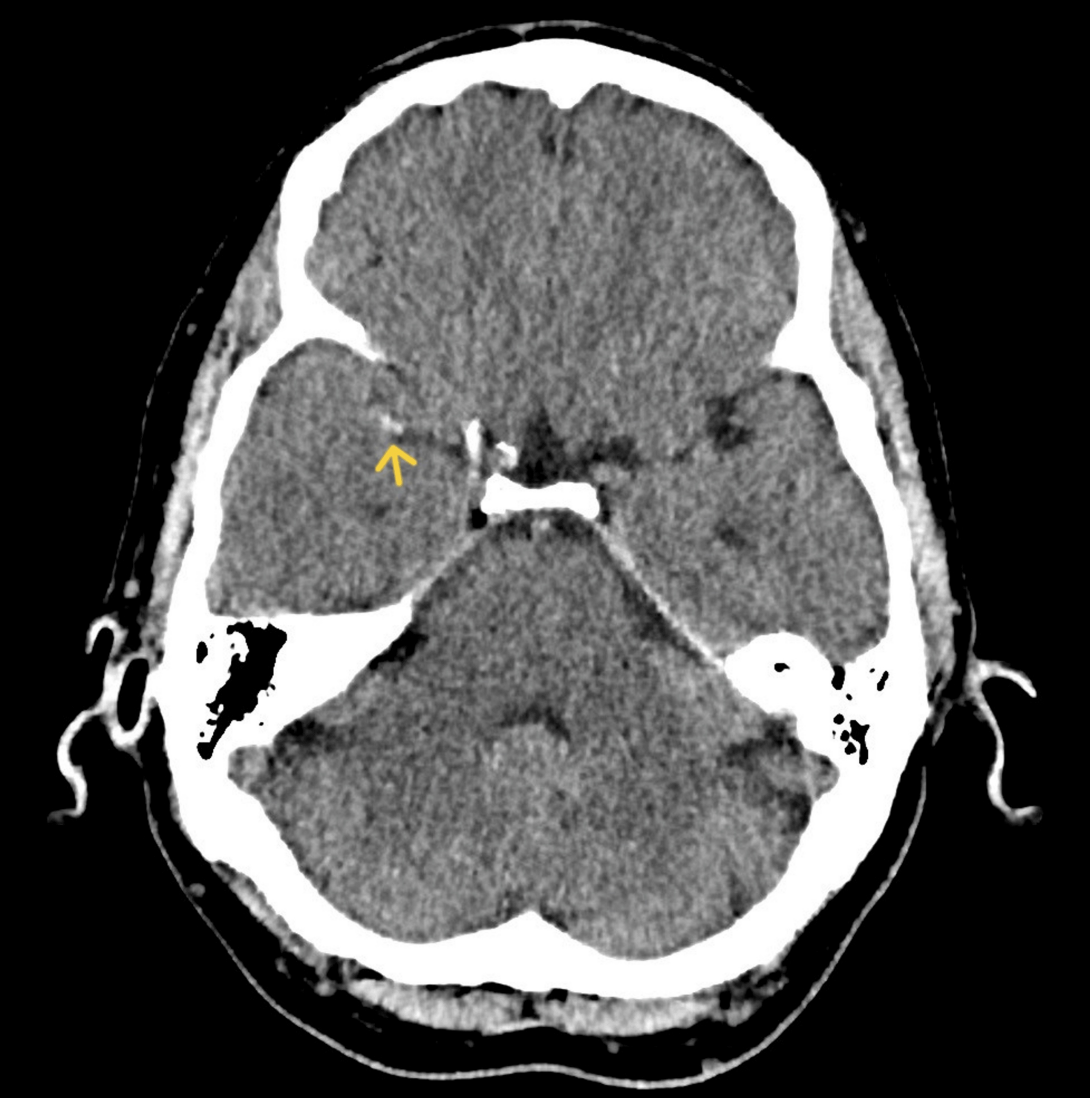
Non-enhanced brain CT scan showing M1 thrombus in the right middle cerebral artery. Yellow arrow pointing to the M1 thrombus.

**Figure 2:**
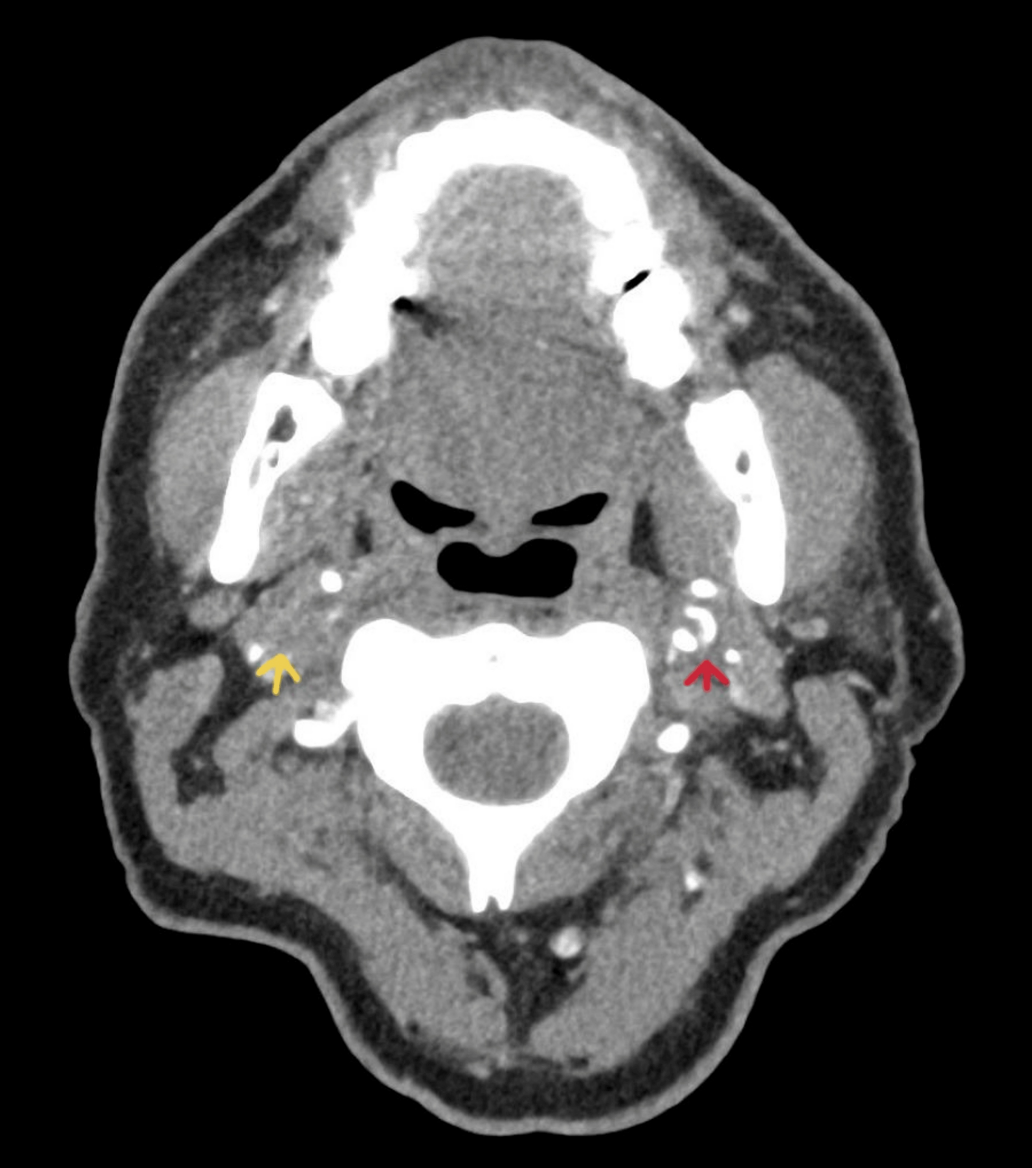
CT angiogram showed a completely occluded right internal carotid artery (yellow arrow), and a severely dissected left internal carotid artery (red arrow) showing true and false lumen.

A non-enhanced brain CT (NCCT) scan showed a small streak of thrombus in the M1 segment of the right MCA (Figure 1), and CT angiography revealed extensive occlusion of the right internal carotid artery (RICA), with visualization only of the proximal 15 mm showing tampered narrowing (flame sign). The radiological findings were consistent with RICA dissection. The supraclinoid portion of the right RICA was visualized and presumed to be reconstituted from collateralization. On the left ICA, the intimal flap showed true and false lumen findings consistent with left ICA severe dissection, with well-opacified left MCA and anterior cerebral artery (ACA) (Figure 2).

The patient was treated with the thrombolytic agent alteplase. After a discussion with the intervention radiologist at a tertiary referral center, it was decided not to consider thrombectomy at this stage as good collateralization was evident from the NCCT. There was initial improvement two hours post-thrombolysis with the NIHSS score decreasing to 1. However, the following day, the patient developed new dense left-sided weakness in both upper and lower limbs, right fixed gaze, left facial weakness, and left-sided inattention. A repeat cerebral angiography revealed a new occluding right M1 thrombus with poor collateral flow within the anterior half of the right MCA territory and early ischemic changes around the insula cortex. This was immediately discussed with the neurosurgery team at the local tertiary referral center, and a decision was made to transfer the patient for thrombectomy. His transfer NIHSS score was 18. He was started on cangrelor intravenously, and thrombectomy was achieved with partial recanalization. The right ICA was recanalized with the insertion of stents. Post procedure, cangrelor was stopped, and the patient was loaded with aspirin 300 mg.

**Figure 3:**
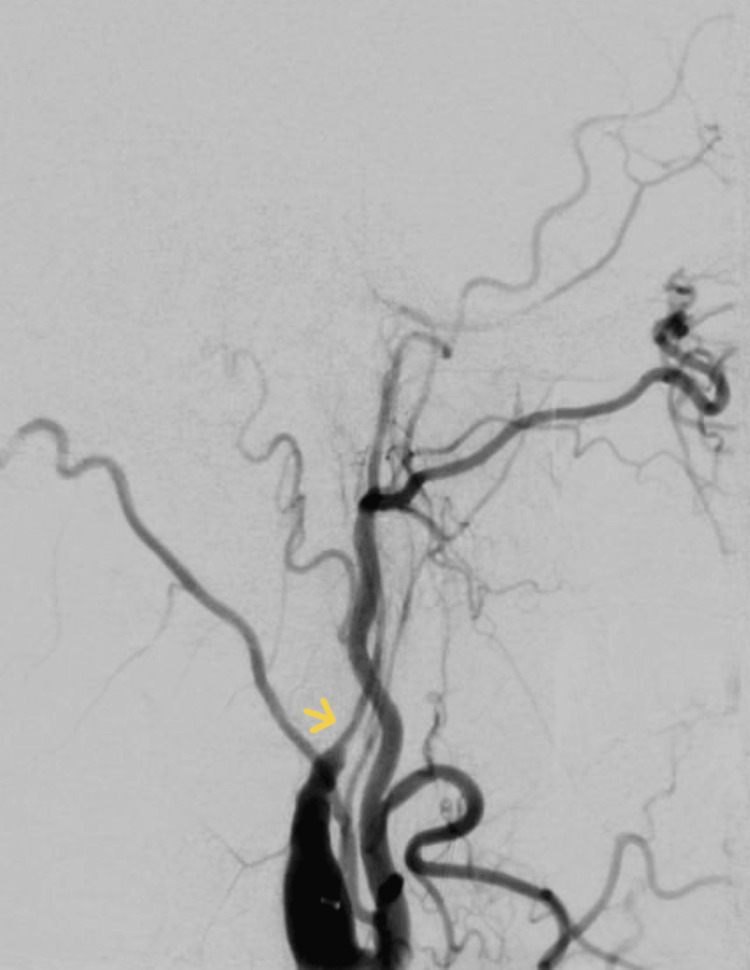
Peri-thrombectomy digital subtraction angiography image showing tapered narrowing of the right internal carotid artery, with findings consistent with dissection.

**Figure 4:**
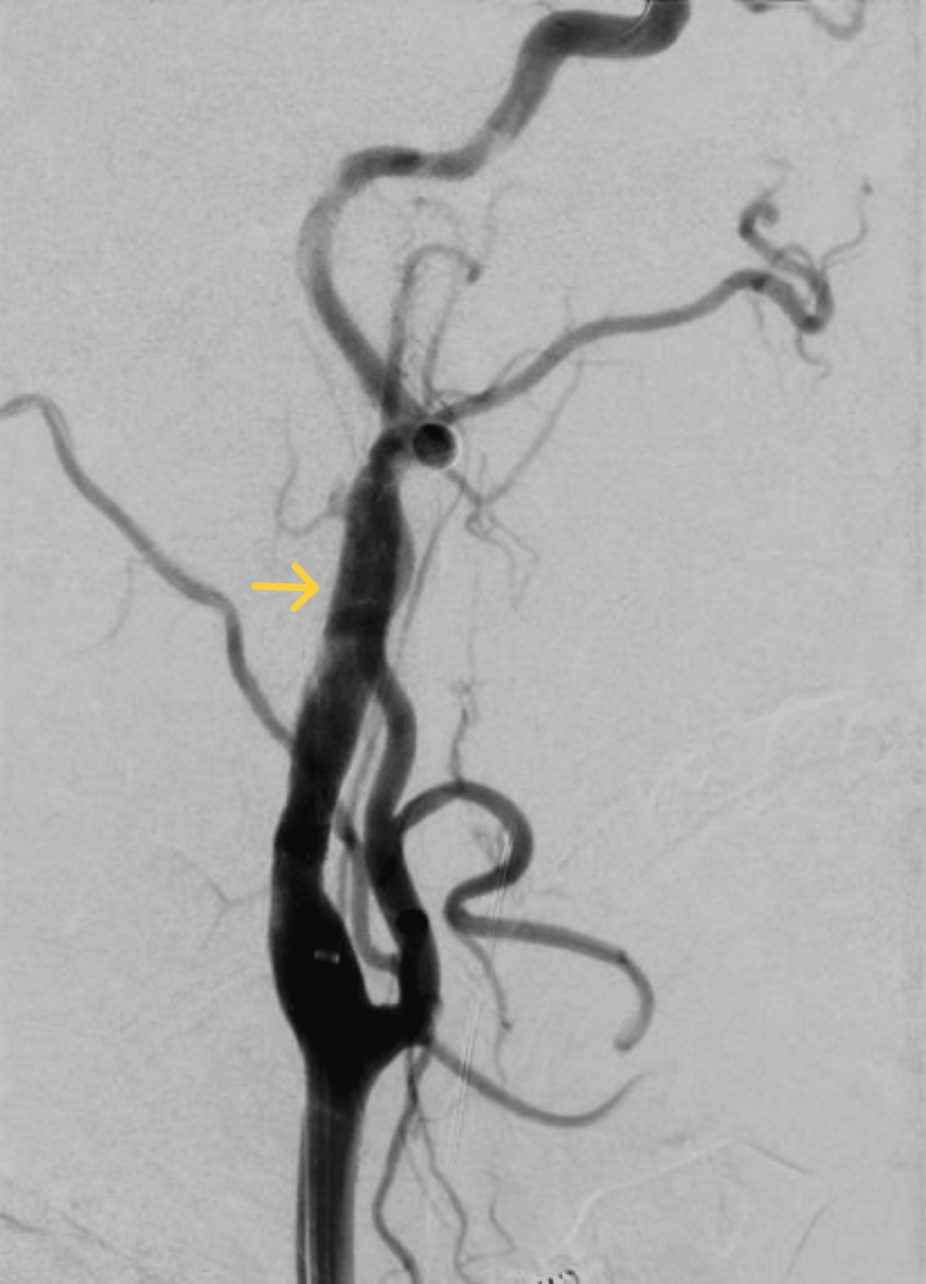
Digital subtraction angiography image post stenting of the right internal carotid artery.

**Figure 5:**
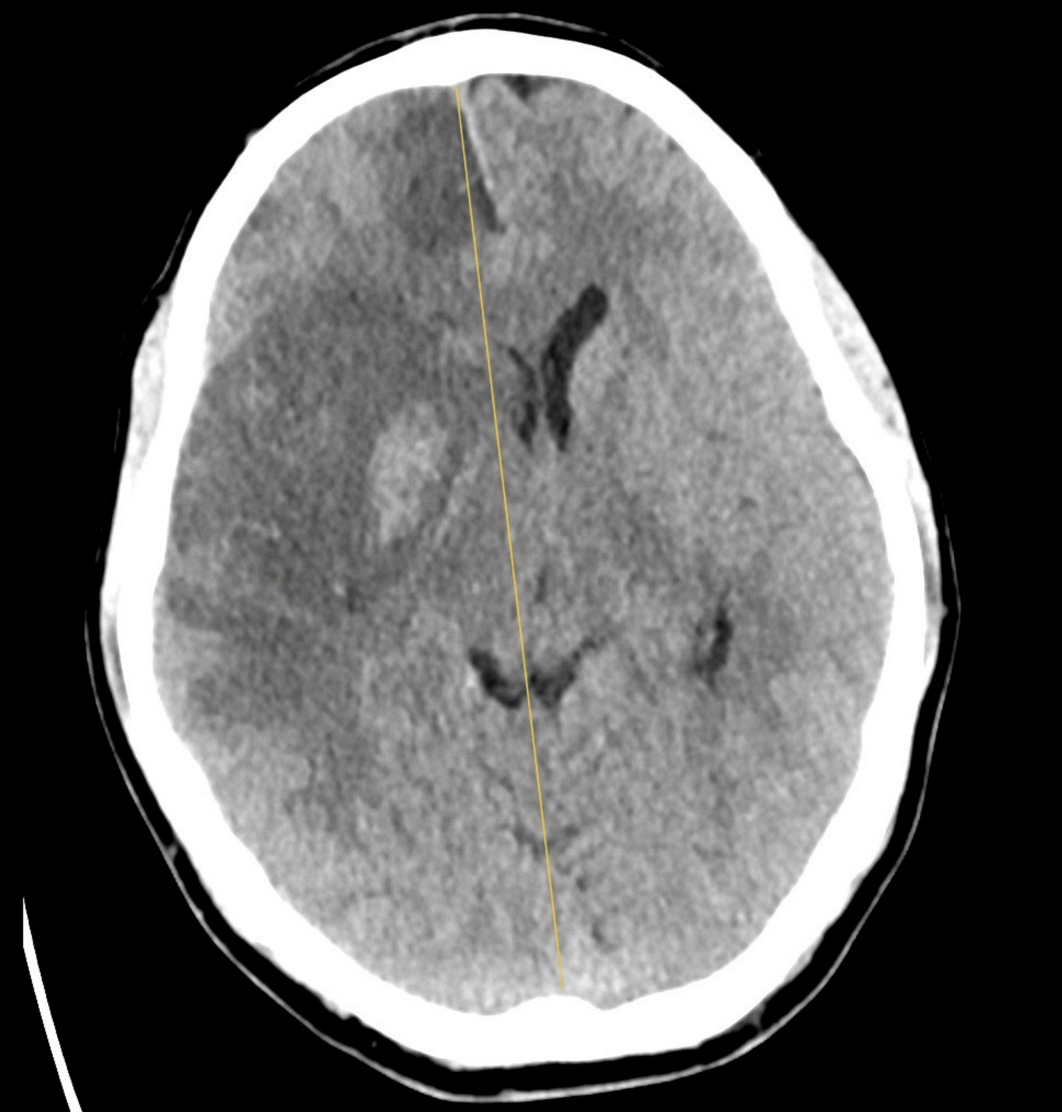
Post-mechanical thrombectomy non-enhanced brain CT showing a large right middle cerebral artery infarct with 8 mm midline shift.

**Figure 6:**
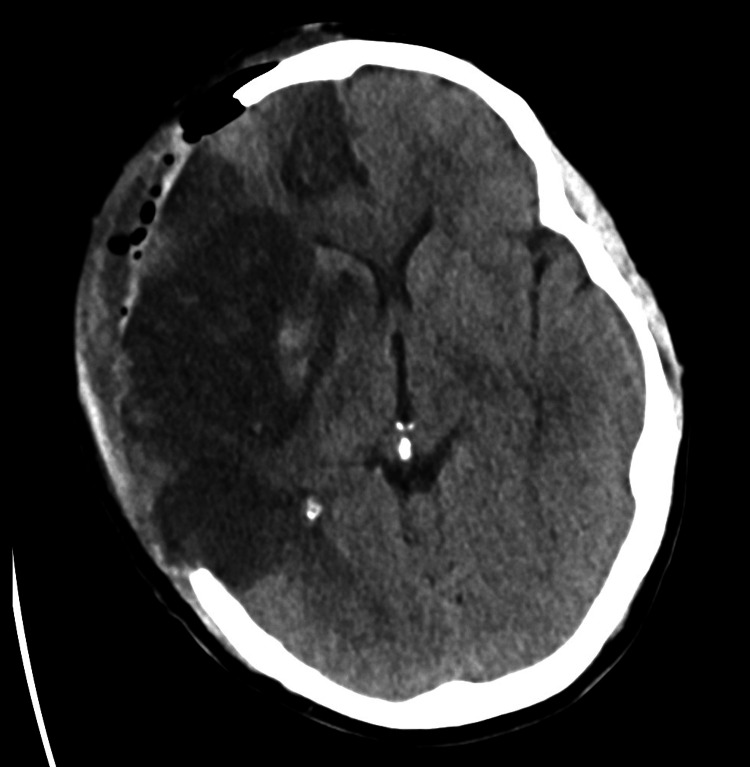
Post-craniectomy non-enhanced brain CT showing a large right middle cerebral artery infarct with resolving mass effect and no hemorrhagic transformation.

Post-thrombectomy NCCT showed a large right MCA infarct involving more than one-third of the right MCA territory with an 8 mm midline shift and mass effect (Figures 3-6). He was monitored overnight, and his Glasgow Coma Scale (GCS) score dropped from 14 to 10, necessitating an emergency right decompressive craniectomy. Post craniectomy, he made a significant recovery with postoperative NCCT showing resolution and no hemorrhagic transformation. At this point, he had a power of 1/5, according to the modified Medical Research Council classification. He was started on antiplatelet monotherapy. A CT angiogram was planned to be done in six months to review left ICA dissection, which was medically managed given no flow obstruction. He was transferred to a stroke rehabilitation unit. After three months, his muscle strength improved to 3+/5 in both upper and lower limbs, and he was able to stand with the assistance of two. He continues therapy in the stroke rehabilitation unit.

Impression

Bilateral internal carotid dissections following a cough complicated with right malignant MCA syndrome treated with right craniectomy.

## Discussion

Epidemiology

Cervical artery dissection (CAD) accounts for 2% of all ischemic strokes but up to 25% in adults under 50, making it a key differential for young stroke patients [1].

CAD can be traumatic or spontaneous. Traumatic dissections result from obvious injuries like whiplash or blunt trauma. Spontaneous dissections can be triggered by minor trauma such as coughing, vomiting, or chiropractic manipulation [4]. Other spontaneous causes include idiopathic factors, often linked to a family history of dissection and connective tissue disorders like Marfan syndrome, Ehlers-Danlos syndrome, and fibromuscular dysplasia. The elongated styloid process, which is eagle syndrome, can also cause spontaneous dissection [5]. Hypertension, migraines, and recent infections are potential predisposing factors for CAD [2,3].

Our patient presented with hemiplegic symptoms and imaging revealed bilateral ICA dissection. This was likely triggered by a minor trauma such as coughing (Valsalva maneuver), though the reason for bilateral ICA dissection remains unclear. ICA dissection from coughing is rare, with few documented cases. It is possible that the patient had an undetected intrinsic vascular weakness or congenital/acquired abnormality. Most CAD patients do not show clinical signs of connective tissue disorders, regardless of the number of arteries affected, including our patient. Although the exact nature of arteriopathy is often unknown, it is believed that patients with spontaneous carotid artery dissection have an underlying structural defect in the arterial wall. Only 1%-5% of these patients have an identifiable heritable connective tissue disorder, and 5% have a family history of spontaneous aortic or its main branch [1].

Clinical diagnosis and investigation of choice

There is significant variation in presentation, ranging from asymptomatic to acute stroke. The usual presentation of CAD includes facial pain, headache (≈65%), neck pain (≈50%), dizziness, and tinnitus, often preceding cerebrovascular ischemic symptoms. Early diagnosis and treatment can reduce the risk of cerebrovascular ischemia [1].

A retrospective statewide study in the United States showed that about one in 30 dissection patients were likely misdiagnosed in the two weeks before their diagnosis, with younger and female patients more prone to missed diagnoses [6].

For suspected CAD, computed tomography angiography (CTA) or magnetic resonance angiography (MRA) are reasonable initial tests. If CTA results are negative but suspicion remains high, MRA with fat-suppressed images is recommended due to its high sensitivity for detecting mural hematomas. Digital subtraction angiography (DSA) should be avoided as a first-line diagnostic tool due to risks like iatrogenic dissection and stroke but may be considered if MRA and CTA are negative [1].

MRA has a high sensitivity for diagnosing carotid dissection (95%) but lower sensitivity for vertebral artery dissection (60%) compared to DSA. CTA, however, has similar sensitivity and specificity to DSA for diagnosing vertebral artery dissection [1].

It is important to check for connective tissue disorders, a possible risk factor for CAD, especially vascular Ehlers-Danlos syndrome (COL3A1 gene) and, to a lesser extent, Marfan syndrome (fibrillin 1 gene), osteogenesis imperfecta (COL1A1 or COL1A2 gene), and Loeys-Dietz syndrome (TGFBR1, TGFBR2, TFFB2, or SMAD3 gene) [1]. Our patient’s blood screen, including immunoglobulins, vitamin B12, folate, hepatitis B and C, HIV, homocysteine, syphilis antibody, and viral antibody screen, showed no cause for his ICA dissection. Tests for prothrombotic conditions, connective tissue disease screen, erythrocyte sedimentation rate, and HbA1c were normal, but random serum cholesterol was 7.3 mmol/L (reference range <5 mmol/L). Interestingly, a meta-analysis shows no association with smoking, diabetes, or hyperlipidemia [7].

Hyper-acute treatment

Intravenous thrombolysis (IVT) using alteplase or tenecteplase is a highly effective treatment for acute ischemic stroke. The European Stroke Organisation recommends considering IVT for patients with acute ischemic stroke with CAD, if they meet other standard criteria, as recommended by current guidelines [1,8]. While IVT carries the risk of intracranial hemorrhage (ICH) and potential enlargement of an intramural hematoma, the proven efficacy of IVT supports its use. In the absence of data indicating significant safety concerns, treatment with IVT remains recommended despite these risks [1].

In patients with large vessel occlusion secondary to CAD, it is recommended to consider emergency mechanical thrombectomy (MT) according to current criteria and guidelines for MT [1].

CAD can cause lumen stenosis or occlusion, though they often do not result in distal hypoperfusion. In cases where hypoperfusion leads to neurological deficits, stenting the stenotic or occluded dissected vessel segment may be considered to enhance distal blood flow and improve outcomes [1].

Our patient on imaging had a right MCA M1 thrombus with good collaterals, meeting the criteria for IVT, hence he was thrombolyzed with alteplase. Although the patient showed initial improvement in his neurological signs, he developed another thrombus causing total occlusion of the M1 artery the next day (15 hours post IVT). The etiology for the second thrombus was thought to be either due to the second thrombus coming from the right ICA dissection or failed IVT. He was transferred for an emergency MT for a large vessel occlusion to the territory center, which was successfully done with stenting of a totally occluded right ICA.

Post-treatment imaging showed a large right MCA infarct involving more than one-third of the right MCA territory with an 8 mm midline shift and mass effect causing malignant MCA infarction. Malignant MCA infarcts occur in about 8% of ischemic strokes and lead to a mortality of around 80% [9]. This is due to the infarction of the MCA leading to acute brain swelling, elevated intracranial pressure (ICP), and brain herniation over a period of five days [9]. As the patient's GCS dropped while monitoring post MT and a repeat CT scan showed features of malignant MCA, he was managed with emergency decompressive craniectomy. Post acute management, our patient recovered with no hemorrhagic complications.

Secondary stroke prevention

A scientific statement published on treatment outcomes in CAD with stroke by the American Heart Association suggests anti-coagulation or anti-thrombotic treatment (dual anti-platelet therapy followed by a single anti-platelet agent or anti-platelet monotherapy) according to the risk factors in each individual case because most ischemic events occur within the first several days after diagnosis [1].

Our patient had a large infarct with a high risk of hemorrhagic transformation, hence he was started on anti-platelet monotherapy. He further had no complications like infractions or bleeding in the next three months of rehab. His power increased with regular rehab from 1/5 on D2 post-craniectomy to 3+/5 in the next three months.

## Conclusions

This case underscores the significance of recognizing that even minor trauma, such as coughing, can trigger bilateral ICA dissections, potentially leading to severe complications. Prompt diagnosis and timely treatment, including IVT and MT in appropriate patients, are crucial for improving functional outcomes. It is essential to consider CAD in the differential diagnoses of young patients presenting with stroke symptoms following trivial trauma, ensuring rapid and effective intervention. Additionally, a CT angiogram should be considered in cases with suspected ICA dissection. The management of complications, such as malignant MCA syndrome, through decompressive craniectomy and subsequent antithrombotic treatment, is also vital in the context of ICA dissection.
